# The Therapeutic Potential of Intra-Articular Injection of Synthetic Deer Antler Peptides in a Rat Model of Knee Osteoarthritis

**DOI:** 10.3390/ijms25116041

**Published:** 2024-05-30

**Authors:** Yu-Chou Hung, Li-Jin Chen, Jen-Hung Wang, Tsung-Jung Ho, Guo-Fang Tseng, Hao-Ping Chen

**Affiliations:** 1 Institute of Medical Sciences, Tzu Chi University, Hualien 970374, Taiwan; 2 School of Medicine, Tzu Chi University, Hualien 970374, Taiwan; 3 Department of Physical Medicine and Rehabilitation, Hualien Tzu Chi Hospital, Buddhist Tzu Chi Medical Foundation, Hualien 970473, Taiwan; 4 Department of Anatomy, School of Medicine, Tzu Chi University, Hualien 970374, Taiwan; 5 Department of Research, Hualien Tzu Chi Hospital, Buddhist Tzu Chi Medical Foundation, Hualien 970473, Taiwan; paulwang@tzuchi.com.tw; 6 Integration Center of Traditional Chinese and Modern Medicine, Hualien Tzu Chi Hospital, Buddhist Tzu Chi Medical Foundation, Hualien 970473, Taiwan; 7 Department of Chinese Medicine, Hualien Tzu Chi Hospital, Buddhist Tzu Chi Medical Foundation, Hualien 970473, Taiwan; 8 School of Post-Baccalaureate Chinese Medicine, Tzu Chi University, Hualien 970374, Taiwan; 9 Department of Biochemistry, School of Medicine, Tzu Chi University, Hualien 970374, Taiwan

**Keywords:** arthritis, chondroprotection, deer antler, joint, kyotorphin, neokyotorphin

## Abstract

Synthetic deer antler peptides (TSKYR, TSK, and YR) stimulate the proliferation of human chondrocytes and osteoblasts and increase the chondrocyte content of collagen and glycosamino-glycan in vitro. This study investigated the peptide mixture’s pain relief and chondroprotective effect in a rat model of collagenase-induced osteoarthritis. Thirty-six adult male Sprague–Dawley rats were divided into three groups: control (saline), positive control (hyaluronic acid), and ex-perimental (peptides). Intra-articular collagenase injections were administered on days 1 and 4 to induce osteoarthritis in the left knees of the rats. Two injections of saline, hyaluronic acid, or the peptides were injected into the same knees of each corresponding group at the beginning of week one and two, respectively. Joint swelling, arthritic pain, and histopathological changes were evaluated. Injection of the peptides significantly reduced arthritic pain compared to the control group, as evidenced by the closer-to-normal weight-bearing and paw withdrawal threshold test results. Histological analyses showed reduced cartilage matrix loss and improved total cartilage degeneration score in the experimental versus the control group. Our findings suggest that intra-articular injection of synthetic deer antler peptides is a promising treatment for osteoarthritis.

## 1. Introduction

Knee osteoarthritis (OA) is a common degenerative joint disease affecting cartilage, bone, synovium, ligaments, periarticular fat, meniscus, and muscle [1,2]. Progressive cartilage degeneration is observed during disease progression and characterized by chondrocyte clustering, irregular surfaces (fibrillations), decreased cartilage volume, and matrix calcification [3,4]. Clinical signs and symptoms include pain, stiffness, reduced joint motion, and muscle weakness [5]. Long-term consequences include reduced physical activity, deconditioning, impaired sleep, fatigue, depression, and disability [6]. The prevalence of knee OA is increasing with the increase in obesity rates owing to dietary changes, lifestyle alterations, and an increase in the aging population [2]. In the population over 60 years old, 10% of males and 13% of females suffer from knee OA, making it one of the leading causes of years lived with disability [7,8]. This has led to significant increases in healthcare and economic costs [9,10,11].

Strong recommendations for knee OA treatment include exercise, weight loss, self-efficacy and self-management programs, Tai Chi exercise, cane use, knee braces, oral and topical nonsteroidal anti-inflammatory drugs (NSAIDs), and intraarticular corticosteroid injections [5,12,13]. Commonly used first-line treatments include NSAIDs such as naproxen and celecoxib; the former should be carefully monitored for side effects such as ulcers and edema, while the latter should be closely watched for the side effects of cardiovascular disease. As for intraarticular injections of drugs, steroid injections are effective for short-term pain relief but may be harmful to cartilage cells, and frequent use is not recommended [1]. The other intraarticularly injected drug, hyaluronic acid (HA), considered as a viscosupplementation for pain and function in patients, currently has an arguable status. In fact, most recent guidelines do not recommend it as a first-line treatment option [1,12,14]. In England, the National Institute for Health and Care Excellence (NICE) guidelines advise against its utilization [15]. Patients with severe symptoms and significant knee cartilage damage may ultimately require knee replacement surgery [1]. Hence, safer and more efficacious pharmacological interventions are urgently needed.

Currently, cartilage protection still awaits a dedicated pharmaceutical solution. This has prompted interest in complementary and alternative medicine [16,17], including nutraceuticals and herbal remedies believed to possess chondroprotective effects and the ability to stimulate cartilage repair [18,19]. The exploration of traditional medicinal systems for potential drugs aiming at reducing inflammation, safeguarding cartilage, enhancing joint functionality, and reinstating activity levels has become a topic of considerable interest. It is interesting to note that crude extracts of deer antlers have therapeutic activities in OA animal models [20,21,22,23,24,25]. However, the active ingredients remain unclear.

Tortoiseshell and deer antler gelatin mixture, known as Guilu Erxian Jiao, taken orally, is a widely used traditional Chinese medicine to treat and prevent osteoporosis and OA without major side effects [26,27]. Modern-day clinical studies have demonstrated that oral ingestion of the mixture increased muscle strength and reduced articular pain and disability in elderly males [28,29]. Our latest biochemical study of the pepsin-digested deer antler gelatin had identified an osteoblast-stimulating pentapeptide, TSKYR (P5; also known as neo-kyotorphin), which can be further degraded by trypsin into dipeptide YR (P2; also known as kyotorphin) and tripeptide TSK (P3) [30]. Both the dipeptide YR and pentapeptide TSKYR had also been found to be analgesic peptides in the bovine brain in other lines of studies [31,32], in which the dipeptide YR acted on a G protein-coupled receptor to stimulate calcium influx [33,34]. Deer antlers and tortoise-shells are rigid, calcium-rich animal tissues that provide calcium, which might work synergistically with P2 and/or P5 to account for the osteoblast proliferation and differentiation effects observed in association with the traditional Chinese medicine Guilu Erxian Jiao [30].

In order to find out whether the identified peptides are the acting components of the traditional Guilu Erxian Jiao, the peptides were biochemically synthesized with over 95% purity [30]. The synthetic peptides were then tested in vitro and found to stimulate the proliferation of cultured human chondrocytes, human osteoblasts, and mouse myoblasts [30,35]. When human chondrocytes were cultured in a three-dimensional configuration, these peptides significantly enhanced the collagen and glycosaminoglycan content as well [35]. The synthetic peptides also inhibited the differentiation of osteoclasts [35]. Taken together, synthetic deer antler peptides could favor bone growth as bone homeostasis is maintained by the counteracting actions of osteoclasts and osteoblasts. Thus, in vitro findings of the synthetic deer antler peptides support the theory that they could be the acting molecules responsible for the musculoskeletal beneficiary effects of Guilu Erxian Jiao in clinics [28,29]. With these, we moved forward in the present study to investigate the therapeutic potential of these synthetic deer antler peptides in experimental animals, namely, we looked into whether synthetic deer antler peptides ameliorate OA in vivo. We employed a rat collagenase-induced knee joint osteoarthritis (CIOA) model and tested the effects of the intraarticular application of synthetic deer antler peptide mixture. The efficacies of the peptide injection in alleviating the symptoms associated with the CIOA were monitored over time, and the chondroprotective effect of the peptides was explored by examining the histopathological changes in the joint at the end of the observation period, 4 weeks after the initial OA induction.

## 2. Results

### 2.1. Body Weight

**Figure 1:**
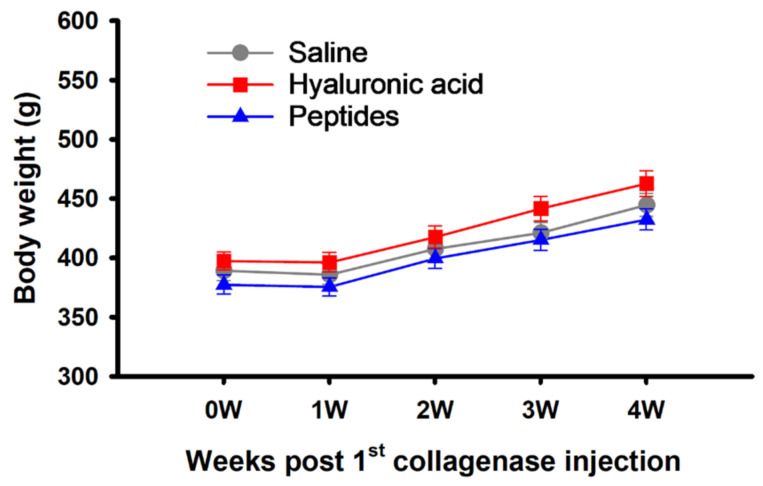
Changes in body weight of synthetic deer antler peptides, hyaluronic acid, and saline treated rats over the 4 weeks of observation. Values are mean ± SEM.

During the week of collagenase injection to induce OA, the body weight of the rats remained unchanged (Figure 1), likely a side effect of the induction process. The animals’ weight increased steadily afterward and to the end of the fourth week of the observation, disregarding subsequent treatments (Figure 1).

In this rat knee OA model, joint swelling and changes in weight bearing and sensitivity of the affected limb were expected; hence, these variables were evaluated from before to 3 weeks after the OA induction as a measurement of the disease progression and physiological responses to the stress.

### 2.2. Knee Diameter Changes

**Figure 2:**
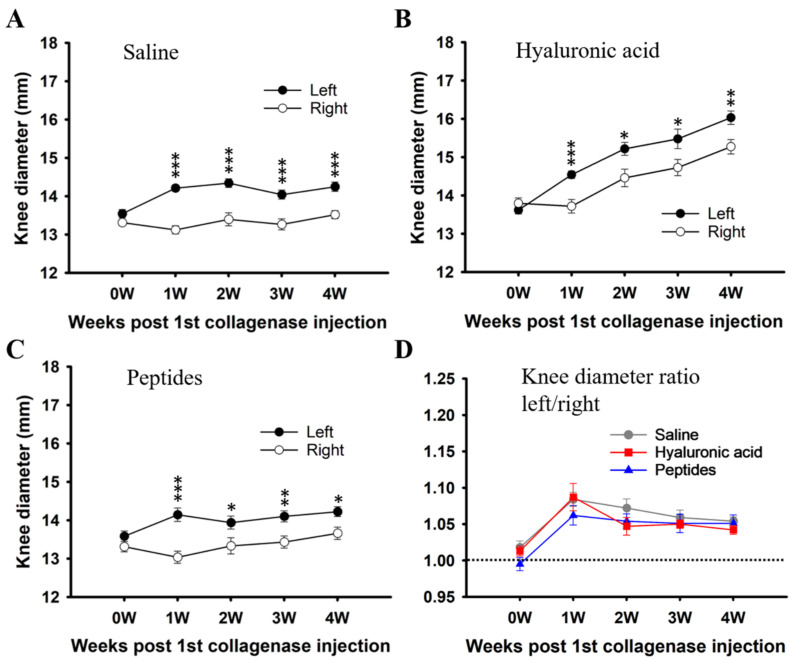
The diameters of the knees of the rats before and after treatments. (**A**–**C**) plotted the diameters of the knees of the affected and contralateral un-manipulated limbs over time of the saline, hyaluronic acid, and peptides-injected groups, respectively. (**D**) plotted the ratio of the knee diameter of the affected limb over the contralateral limb to show the swelling. Values are mean ± SEM. * *p* < 0.05, ** *p* < 0.01, *** *p* < 0.001.

CIOA led to a significant increase in the diameter of the injected knee by the end of the first week in all three groups of rats studied (Figure 2A–C). Enlargement of the affected knee persisted to the end of the fourth week in all three groups of rats, disregarding subsequent treatments (Figure 2A–C).

To take the small difference in the knee diameters between the three groups of rats into account, the ratio of the diameter of the injected over that of the non-injected knee of each animal of all three groups of rats is derived and plotted in Figure 2D. An increase in ratio indicates an enlarged or swollen knee. In the saline group (filled circle, Figure 2D), i.e., the CIOA with presumably no effective treatment, the ratio peaked at the end of the first week, i.e., 3 days after the second collagenase injection. It then dropped slightly by the end of the second week, and decreased slowly but steadily to the end of the fourth week; it, however, remained higher than the no-difference value (dotted line, Figure 2D). For the HA as well as the peptides treatment groups, changes in this ratio, by and large, followed that of the saline group and there was no difference in this ratio between these three groups of rats from the first to the fourth survival weeks (compared between filled triangle, filled square, and filled circle, Figure 2D). Neither peptides nor HA treatment returned the knee diameter of the CIOA limb to the same as that of the non-CIOA limb by the end of the fourth examination week (Figure 2D).

### 2.3. Weight-Bearing

**Figure 3:**
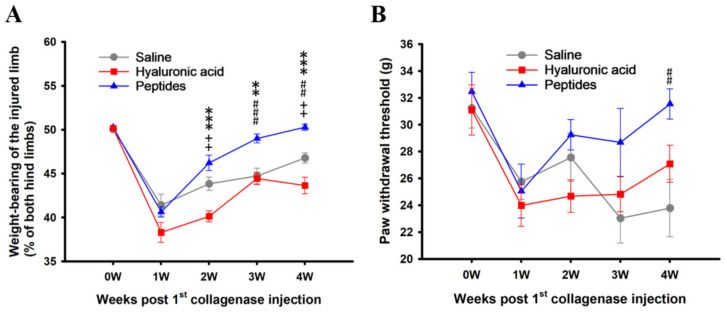
Effects of synthetic deer antler peptides injection on collagenase-induced knee joint osteoarthritis (CIOA) in weight-bearing and paw withdrawal threshold tests. (**A**) the weight-bearing capacity, as a ratio of both hind limbs, of the treated limb before and after CIOA and following treatments. (**B**) the paw withdrawal threshold of the studied limb before and after treatments. Values are expressed as mean ± SEM. ** *p* < 0.01, *** *p* < 0.001, peptides vs. hyaluronic acid; ## *p* < 0.01, ### *p* < 0.001, peptides vs. saline; ++ *p* < 0.01, saline vs. hyaluronic acid.

Following CIOA, a significant decrease in the weight-bearing capacity of the arthritic hind limb was observed in all three groups of rats examined at the end of the first week, i.e., 3 days following the second collagenase injection (Figure 3A). There was no difference between the three groups at this time point, consistent with the fact that peptides or control treatment were administered right after this. There was a gradual increase in the weight-bearing ratio of the left hind limb in all three groups from the second to the end of the fourth week, disregarding peptides, HA, or saline treatment. However, the recovery of the peptides group was significantly better than that of the HA group at the end of the second week, i.e., one week after peptides treatment (***, *p* < 0.001 Figure 3A), and significantly better than both HA and saline groups at the end of both the third (** *p* < 0.01, ### *p* < 0.001 respectively, Figure 3A) and fourth examination weeks (*** *p* < 0.001, ## *p* < 0.01 respectively, Figure 3A). In fact, by the end of the fourth examination week, the weight-bearing capacity of the left hind limb of the peptides group had returned to 50.3 ± 0.4% of both hind limbs (Figure 3A), indicating that it regained the full weight-bearing capacity as the un-manipulated right limb. The weight-bearing capacity of the saline group also appeared better than that of the HA group, especially at the end of the second and fourth weeks (++ *p* < 0.01 in both cases, Figure 3A).

### 2.4. Mechanical Allodynia

CIOA resulted in a marked decrease in the paw withdrawal threshold (PWT), an indication of mechanical allodynia, in the affected left hind limb of rats scrutinized at the end of the first week of the experiment, i.e., 3 days after the two doses of collagenase injections (Figure 3B). Again, there was no difference in the PWT between the three groups of rats before testing the therapeutic effects of peptides versus HA and saline treatments (Figure 3B). Intraarticular peptides administration markedly reversed the decrease in the PWT of the injected limb one week after treatment, and returned to a level equivalent to that before CIOA induction by the end of the fourth examination week, i.e., 3 weeks following peptides treatment (##, *p* < 0.01, Figure 3B). On the other hand, HA and saline treatments failed to consistently reverse the PWT changes throughout the 3 post-treatment weeks examined (Figure 3B).

### 2.5. Histopathology and Histopathological Scoring

**Figure 4:**
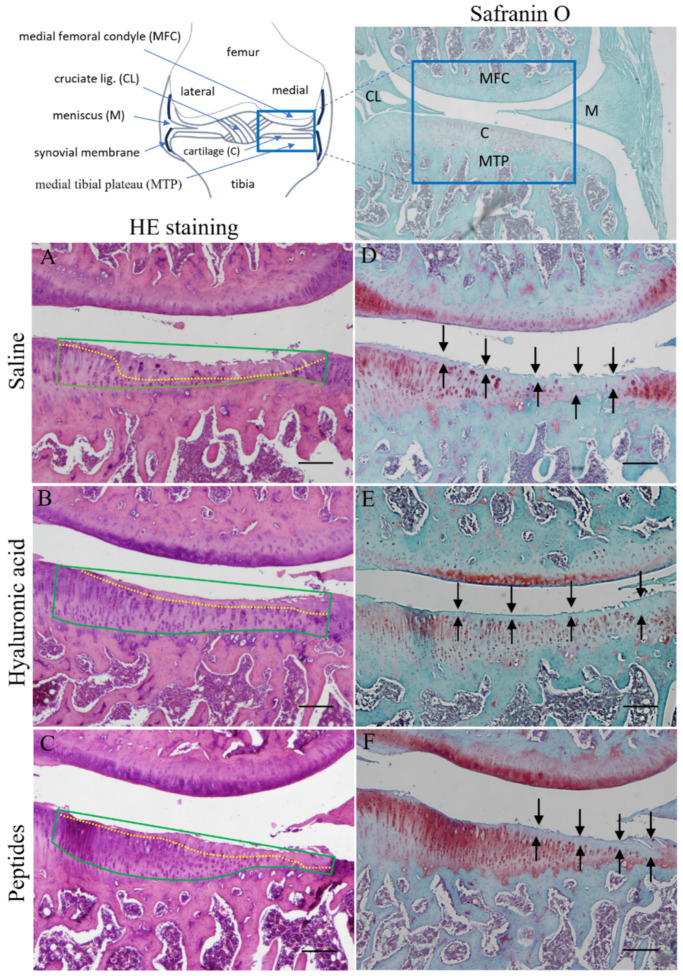
Effects of synthetic deer antler peptides on the joint structure of the affected knee. A schematic drawing of the knee joint of a rat with the main structures labeled is illustrated in the upper left panel. The square area in the upper right panel of an unaffected knee joint stained with Safranin O corresponds to the rectangular area shown in the upper left panel. The middle portion of the medial tibial plateau (MTP, 1500 μm in width) directly bearing the weight shows the most severe lesion in collagenase-induced osteoarthritic rats, therefore, the area selected for analyses. The area of non-viable cartilage characterized by significant chondrocyte loss but with collagen retention (area above the yellow dashed line) lies closer to the joint cavity. The green line delineated area represents the area of the cartilage before erosion (**A**–**C**). Representative joint histopathology of the three treatment groups as labeled shows a more severe and extensive degeneration in the MTP of the saline group as compared to that of the hyaluronic and peptide groups (**D**–**F**). Loss of proteoglycan in the MTP was evident (between the upper and lower rows of black arrows) in the adjacent sections of the joint stained with Safranin O (reddish staining) in each of the corresponding treatment groups illustrated. The staining roughly corresponds to the area with chondrocytes retaining. The MTP of the peptides-treated OA joint shows more reddish staining than both the HA and the saline-treated. Notice that all the sections illustrated were from the tissue following decalcification. Scale bar = 200 μm.

Cartilage degeneration, including erosion, fissures, cell depletion, and weak Safranin O staining of the cartilage matrix, was observed in all three groups, with the most severe and extensive degeneration observed in the saline group (Figure 4). The middle portion of the medial tibial plateau (MTP), likely directly underneath the weight loaded onto the tibia, shows the most severe lesion in the collagenase-induced osteoarthritic joints. The concave upper contour of the remaining cartilage of the MTP of all three treatment groups, especially the saline and HA-treated, as opposed to the flat to slightly convex contour of that of the non-CIOA joint (upper right panel, Figure 4), supports the theory that this area bears most of the weight of the joint and the concave surface represents erosion following CIOA. A 1500-μm-width strip of this MTP cartilage was thus selected for analysis. The area of the MTP above the tidemark to the straight line connecting the two upper corners on each side (green line delineated area) represents the arbitrary original cartilage area in the MTP of the respective joint illustrated (Figure 4A–C). The substance-free area below this arbitrary line is where the presumed eroded part of the cartilage was located. Below this is the non-viable cartilage, characterized by significant chondrocyte loss but with collagen retention (area above the yellow dashed line, Figure 4A–C). The non-viable part of the cartilage lies closest to the joint cavity and above the chondrocyte-retention part of the cartilage in this stage of the CIOA.

The saline group shows more severe and extensive degeneration in the MTP as compared to that of the HA and peptide groups (compare A with B and C, Figure 4). Safranin O is a basic dye that stains the cartilage (proteoglycans, chondrocytes, and type II collagen) with varying red shades. Safranin O staining of the adjacent section of each corresponding joint shows markedly less staining, presumably a loss of proteoglycan, in the MTPs of both the saline and HA groups (between the upper and lower rows of black arrows, Figure 4D–F). The staining area roughly coincided with the area of chondrocytes-retaining cartilage. Overall, treatment with synthetic deer antler peptides or HA reduced cartilage degradation. However only the peptides treated showed better preservation of proteoglycans (compare F with D and E, Figure 4). By the end of the fourth observation week, none of the groups exhibited hypertrophy of the synovial membrane or infiltration of inflammatory cells into the synovial membrane. Besides the cartilage, no sign of subchondral bone ossification or marginal osteophyte was observed in any of the joints studied.

**Figure 5:**
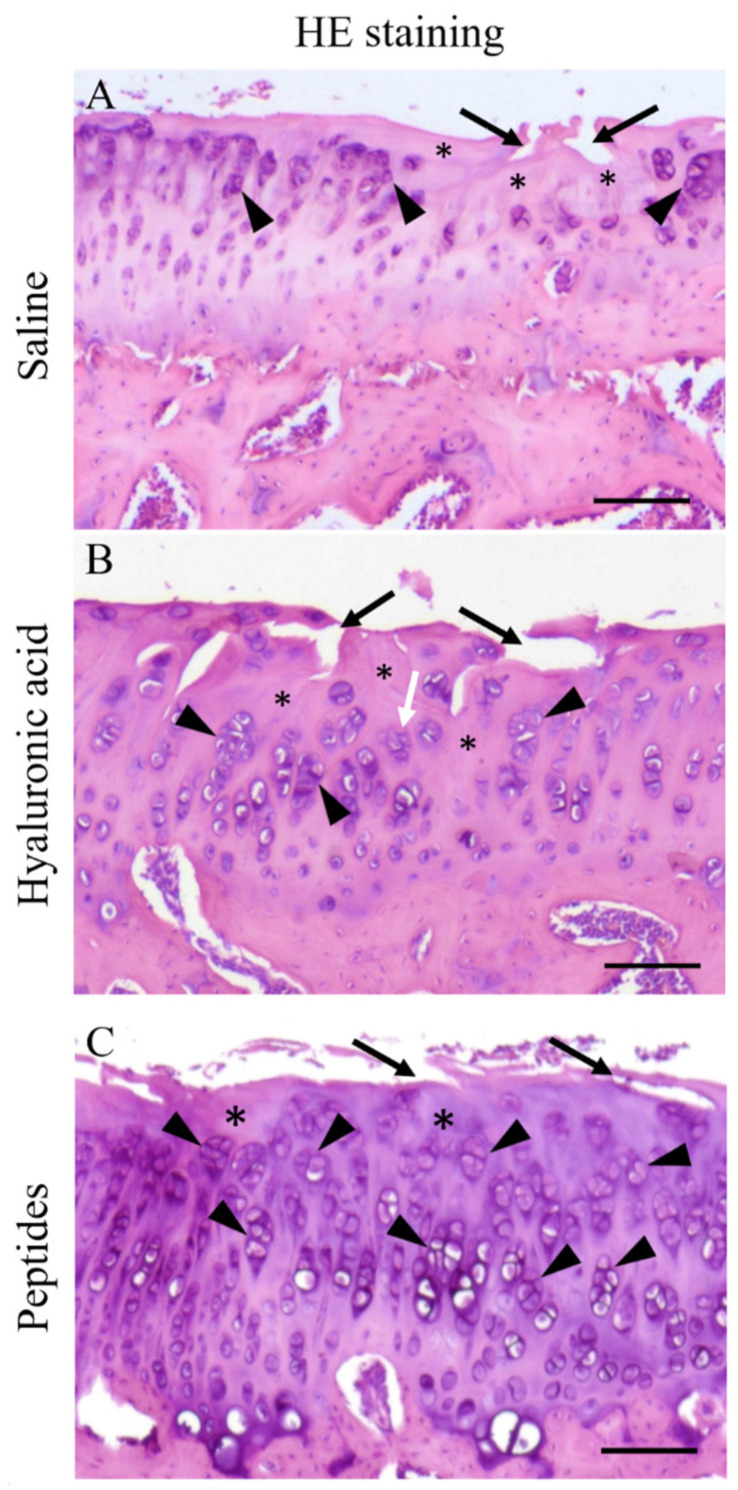
Effects of synthetic deer antler peptides on the cartilage of the arthritic joints. Extensive cartilage degeneration was characterized by areas of fissure and erosion (black arrows; **A**–**C**) and cell depletion (*; **A**–**C**). Chondrocyte clustering and hypertrophy are shown with black arrowheads. All sections illustrated were from the affected knee joints following decalcification. Scale bar = 100 μm.

Figure 5 shows an enlarged view of a representative portion of the MTP of the joint of each treatment group. Extensive cartilage degeneration, characterized by areas of fissure and erosion (black arrows, Figure 5A–C) and cell depletion (*, Figure 5A–C) were evident in the joint cartilages of all three treatment groups. In addition, the joint cartilages of all three groups also show clustering and hypertrophy of chondrocytes (arrowheads, Figure 5).

**Figure 6:**
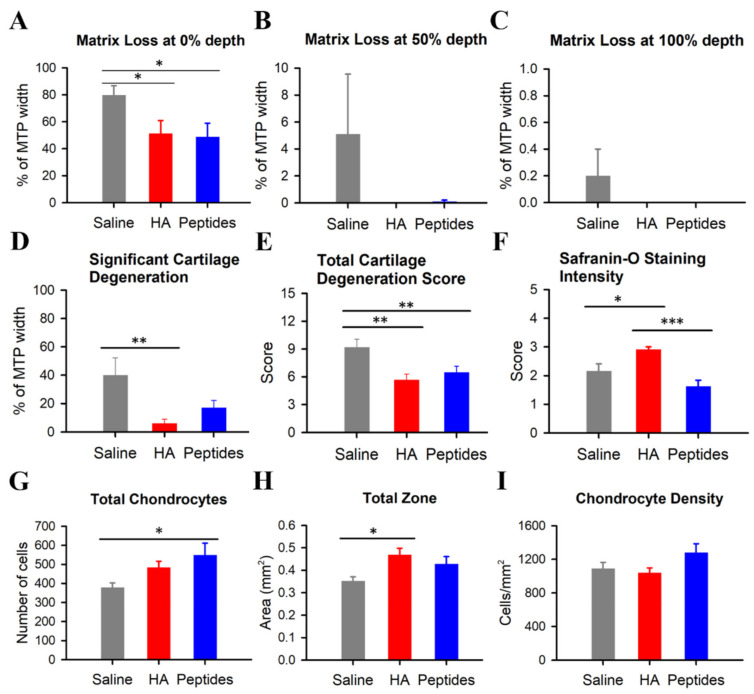
Synthetic deer antler peptides demonstrated comparable efficacy to hyaluronic acid (HA) in reducing cartilage damage, as indicated by histopathological scorings. Evaluated parameters include cartilage matrix loss width (CMLW) along the surface (0% depth, panel (**A**)), at the level of the mid zone (50% depth, panel (**B**)), and at the tidemark (100% depth, panel (**C**)). (**D**) significant cartilage degeneration width (SCDW); (**E**) total cartilage degeneration score (TCDS); (**F**) Safranin O staining intensity score (according to MANKIN, please see Supplementary Information for further details). Notice that higher score means less staining; (**G**) total number of chondrocytes; (**H**) total zone (total area of cartilage); (**I**) chondrocyte density in the total cartilage area. Values are mean ± SEM. * *p* < 0.05, ** *p* < 0.01, *** *p* < 0.001.

Joint cartilage matrix loss following CIOA was evaluated at a 0% (surface), 50% (mid-zone), and 100% (deep to the tidemark) depth from the presumed original upper border of the joint cartilage (a straight line connecting the two upper corners at the two lateral ends of the MTP) to the tidemark. The width of the MTP cartilage showing matrix loss as a percentage of the total MTP width was high, particularly at the 0% depth, in all three treatment groups (Figure 6A). Matrix loss at the 50% and 100% depth was prominent in the saline group but was minimal in the peptides and HA groups (Figure 6B,C). When comparing the percentage of the MTP showing cartilage matrix loss at the 0% depth, a significant difference was detected in the saline group, an average of 80 ± 7%, as compared to those of the peptides and HA groups (*p* < 0.05, Figure 6A). Synthetic deer antler peptides and HA reduced the cartilage matrix loss from the superficial to the deep zone (Figure 6A–C, red and blue histograms) as compared to that of the saline group (gray histogram). The percentages of the MTP of the HA and peptides groups at the 50% and 100% depth showing cartilage matrix loss were so low that statistical comparison between them and the saline group is not legitimate.

We, in addition, looked into the overall joint cartilage degeneration status of the three treatment groups based on the above histological sections. In this, the significant cartilage degeneration width (SCDW) represents the width of the tibial cartilage compromised by at least 50% of its thickness owing to the absence of 50% or more chondrocytes, with or without matrix loss. The SCDW width of the MTP cartilage as a fraction of the total width of the MTP of the three treatment groups is plotted in Figure 6D. The saline group has a much wider SCDW width, 40 ± 12% of the total MTP width, as compared to the 6 ± 3% of the HA group (*p* < 0.01, Figure 6D). The percentage of MTP of the peptides group with SCDW was 17 ± 5%, suggesting a trend towards protection against cartilage degeneration as compared with that of the saline group.

The other estimate of the joint cartilage condition is the total cartilage degeneration score (TCDS), an overall evaluation of cartilage pathology. It was higher in the saline group (9.2 ± 0.9), compared to the peptides group (6.5 ± 0.7) and HA group (5.7 ± 0.6), signifying a more extensive matrix and chondrocyte loss alongside moderate degeneration in the saline group (*p* < 0.01, Figure 6E). Both the peptides and HA groups demonstrated reduced cartilage degeneration and chondrocyte loss compared with the saline group. These findings suggest that intraarticular injection of synthetic deer antler peptides yields an effect comparable to that of the HA in reducing cartilage damage.

Figure 6F plots the Safranin O intensity score (according to MANKIN, please see Supplementary Information) of the MTP of the joints of the three treatment groups. Higher score means more severe loss of Safranin O staining. The score was significantly higher in the HA group (2.9 ± 0.1) compared to those of the saline (2.2 ± 0.2) and the peptides groups (1.6 ± 0.2), indicating more severe proteoglycan loss in the HA than the saline and the antler-derived peptides groups (*p* < 0.05 and *p* < 0.001, respectively, Figure 6F).

The analysis also showed a higher chondrocyte count in the MTP of the peptides group than that of the saline group (*p* < 0.05; Figure 6G). There was a significant disparity in the preserved cartilage area (total zone) between the saline and HA groups (*p* < 0.05; Figure 6H). Chondrocyte density assessment involved quantifying chondrocytes within the above designated zones. It nevertheless revealed no significant differences between the three treatment groups (Figure 6I).

## 3. Discussion

In this study, deer antler peptide mixtures (P5, P3, and P2) appeared to have a pain-relieving effect, as shown in the weight-bearing test and paw withdrawal test results. Notably, the weight-bearing capacity returned to 50 ± 1% by the end of the fourth week of the test, which meant that there was no difference in weight-bearing between the affected limb and the unaffected limb after the induction of OA. This pain-relieving effect of intraarticular synthetic peptides application in live animals is in line with the report of a notable reduction in arthritic pain in an in vivo study focusing on the effects of tortoise- shell and deer antler gelatin extracts administered orally over 28 consecutive days on degenerative OA [20,36]. Thus, this experimental animal study provides evidence that these synthetic peptides are likely the acting components of the tortoiseshell and deer antler gelatin in relieving joint pain.

In the present study, we used HA as a positive control. Surprisingly, no significant difference in mechanical allodynia relief was revealed between the HA and saline groups. In addition, the HA group displayed more delayed weight-bearing ability recovery than that of the saline group. These findings are consistent with the results from an increasing number of recent clinical studies showing that viscosupplementation is associated with a clinically irrelevant reduction in pain intensity [15].

Additionally, our histological examination shows that intraarticular injection of the synthetic deer antler peptides mixture attenuated the erosion on the superficial layer of the joint cartilage and reduced the degeneration of the chondrocytes in the joint cartilage in several parameters examined, including matrix loss at a 0% depth and the TCDS. Furthermore, matrix loss at a 50% and 100% depth, which was prominent in the saline group, was minimal in the peptides, as well as the HA group. In other words, peptides and HA have similar effects in reducing deep matrix loss of the joint cartilage. The TCDS assesses the overall cartilage degeneration, with chondrocyte loss as the primary determinant of the score. In this regard, both the peptides and HA-treated groups were significantly better than the saline control group. Thus, based on this scoring system, both peptides and HA treatments provide comparable protective effects against joint cartilage degeneration.

When looking specifically into the number of chondrocytes in the residual joint cartilage, in this study the total chondrocyte count of the peptides-treated was significantly higher than that of the saline control. However, there was no discernible difference in chondrocyte density between all three treatment groups, likely a result of the fact that both peptides and HA-treated groups preserved a larger matrix area than that of the saline group. This raised the issue of whether antler peptides directly stimulate chondrocyte proliferation or stimulate chondrocyte proliferation following injury [37], which remains to be clarified in future experiments.

Besides affecting chondrocytes, our earlier study also showed that synthetic deer antler peptides increased the collagen and glycosaminoglycan levels in a three-dimensional chondrocyte culture [35]. In line with this, this in vivo study showed higher proteoglycan content in the joint cartilage of the synthetic peptides than that of the saline group, supporting a positive impact of the synthetic peptides on the proteoglycan homeostasis during OA progression. On the other hand, our results that HA treatment led to reduced proteoglycan levels in this in vivo model are consistent with the suggestion from previous studies that HA treatment might result in a negative long-term impact on the injured joint, i.e., it has a potential role in hastening joint degeneration [38,39].

About the effects of synthetic deer antler-derived peptides on bone formation, our previous culture study reported that it stimulated osteoblast proliferation and bone formation (particularly in conjunction with calcium) [30]. Nevertheless, in the present study we failed to see any apparent histopathological sign of subchondral ossification or marginal osteophyte formation in live animals. Neither did the HA or the saline group of rats show any sign of bone formation as well. Causes for the lack of a bone-formation-supporting effect of the synthetic antler peptides in the present model include: first, the lack of a sufficient concentration of calcium ions in the synovial fluid at the time of peptides injection might have hindered osteoblast proliferation; second, bone formation could have a time frame beyond our observation period since previous animal experiments reported that osteophyte formation might be observed four weeks after collagenase injection and it typically became more prominent by the sixth week [40]. This raises the need for future experiments to look into the long-term effects of intraarticular synthetic antler peptides treatment.

It is generally agreed that there is a complex interaction between the cartilage and subchondral bone in OA; however, the exact initiating factor and underlying mechanism remain debatable [41,42]. The anterior cruciate ligament transection model suggests that cartilage damage likely occurred as a consequence of subchondral bone remodeling. Subchondral bone remodeling, on the other hand, appears to result from cartilage degeneration in the CIOA model [42]. In our study, intraarticular injection of synthetic deer antler peptides attenuated cartilage erosion, which might thus preclude the subchondral bone remodeling cascade and, hence, provide a new potential medication in clinical OA treatment. In this study, we observed no infiltration of inflammatory cells into the synovial membrane at the end of our 4-week observation period. Although no direct inflammatory parameter measurements were included in the present study, the results of the improved weight-bearing capability of the affected limbs and decreased mechanical allodynia after synthetic peptides treatment argued for a decreased inflammatory process following synthetic deer antler peptides treatment.

Taken together, the present results support the theory that synthetic deer antler peptides mixture applied intraarticularly has a joint-degeneration-alleviating effect and a chondroprotective effect on the joint cartilage, hence, a therapeutic effect in our animal model of knee OA. The results, however, are based on a collagenase-induced, i.e., less naturally occurring case of OA, over a short time course. Further experiments on a cause-wise, more clinically relevant case of OA are warranted. Furthermore, more work is still needed to find the optimal concentration of each component of the synthetic deer antler peptides alone and in a mixture. Additional experiments are needed to clarify whether calcium can improve the outcome of the peptides treatment as well. Despite all these loopholes, the fact that the synthetic deer antler peptides that we used in the present study have been shown to be compatible with human chondrocytes and osteoblasts and stimulated their proliferation and growth in vitro [30,35] argues that they are likely to be compatible with human tissue in future clinical trials. Thus, evidences from the present live animal and earlier culture studies open up a promising potential for the synthetic deer antler peptides mixture or its variants to be tested in human OA patients in the near future.

## 4. Materials and Methods

### 4.1. Animals

Thirty-six adult male 10-week-old Sprague–Dawley rats (388 ± 28 g body weight on the day of OA induction; Lasco, Ilan, Taiwan) were included in this study. The number of animals used (sample size) was calculated with G*Power 3.1.9.2 with the effect size of 0.44, α of 0.05, power(1-β) of 0.80, number of groups as 3, number of measurements as 4, and correlation among repeated measures as 0.5. This yielded an estimated sample size of 36, i.e., 12 rats per group. Young male rats were used to avoid running into the potential tissue-protecting effect of estrogen or its metabolites and the cyclic fluctuation nature of this sex hormone in the females. All animals were caged in a room with a constant temperature (24 ± 1 °C), humidity (60–65%), light-controlled (12/12 h light-dark-cycle) with food and water ad libitum and allowed to acclimatize for a week before experiments. All procedures were approved by the Institutional Animal Care and Use Committee of Tzu Chi University (approval number: 11106). All efforts were made to minimize animal suffering such as during handling.

### 4.2. Study Design

**Figure 7:**
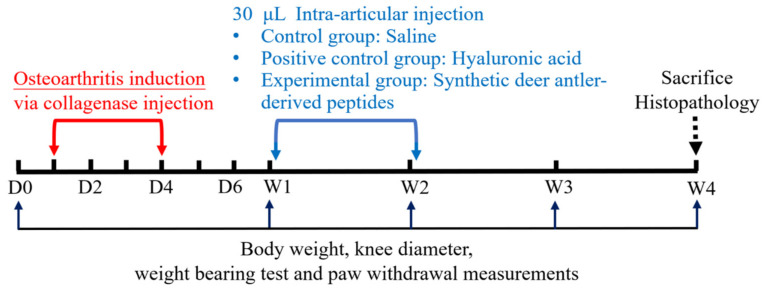
Schematic timeline of the present study design. Rats received a collagenase injection into the left knee on days 1 and 4 to induce osteoarthritis and were divided into control, positive control, and experimental groups to receive corresponding treatment in the first and second weeks. Rats were sacrificed 4 weeks after the first collagenase injection for histology of the joint. Assays were conducted at five time points over 4 weeks. Notice that the time abscissa is not to scale. D, number of days; W, weeks.

Figure 7 is a schematic diagram of the design of the present study, which stretched over the course of 4 weeks. Rats received two injections of 30 μL of 500 U of type II collagenase into the left knee, one on day one and the other on day four, to induce OA. The animals were then divided into three, the control, positive control, and experimental groups. In current clinical practice, an intraarticular hyaluronic injection was administered once a week. Therefore, the control group received intraarticular injections of 30 μL saline into the collagenase-treated knee once a week for 2 consecutive weeks following the initial collagenase injection; the positive control group was treated with two 30 μL HA injections following the same time course; the experimental group was treated with two injections of 30 μL synthetic deer antler peptides (P5, P3, and P2, 20 μg each) in saline. The rats were sacrificed 4 weeks after the first collagenase injection. The four assays listed below the time bar were performed at the five time points indicated in the figure.

### 4.3. Induction of OA and Subsequent Treatments

Under brief isoflurane anesthesia (5% for induction and 2% for maintenance), intraarticular injections were administered using a Hamilton syringe equipped with a 26 G needle inserted through the patellar ligament into the left knee joint. All animals received two injections, one on day one and another on day four. The injection fluid consists of 30 μL of 500 U of *Clostridium histolyticum* type II collagenase (Thermo Fisher Scientific, Waltham, MA, USA) dissolved in physiologic saline and filtered through a 0.22 μm membrane according to the method of Lee et al. [43]. The right knee was left untreated.

About the synthetic deer antler peptides, P3 and P2 have been shown to have the most substantial chondrocyte-proliferation-stimulating effect [30]. Degradation of the injected peptides might occur in the synovial fluid. Cleavage of P5 might provide additional P3 and P2. Moreover, our earlier in vitro study revealed an increase in glycosaminoglycan and collagen content in the human chondrocyte culture in the presence of P5, P3, and P2 [35]. Taking all of these into consideration, in this experiment, the three synthetic deer antler peptides, P5, P3, and P2 (purity > 95%; synthesized by Mission Biotech (Taipei, Taiwan), were prepared in equal amounts in a mixture for intraarticular injection. One week following the initial collagenase injection, the animals were stratified into three groups: group 1 (n = 12) the control group, received intraarticular injections of 30 μL saline into the collagenase-treated knee once a week for 2 consecutive weeks; group 2 (n = 12), the positive control group, received two 30 μL of 10 mg/mL HA (ARTZ Dispo, Seikagaku, Tokyo, Japan) with a molecular weight of approximately 900 kDa [44] in similar time points as the first group; group 3 (n = 12) received two intraarticular injections of 30 μL of synthetic deer antler peptides mixture in saline containing 20 μg of P5, P3, and P2 each following the same schedule (please see also Figure 7). After each injection, several flexions and extensions of the joint were performed to ensure a homogeneous distribution of the injected material within the joint cavity. Body weight measurements were recorded before the first collagenase injection and at the end of the first, second, third, and fourth week after the first collagenase injection (Figure 7).

### 4.4. Joint Swelling Evaluation

Joint swelling was evaluated weekly using a caliper to measure the articular diameter in millimeters at the timing indicated in Figure 7. A caliper was positioned along the articular line to determine the transverse diameter of the left knee joint. The unaffected knees of the animals were also measured for comparison. Each knee was measured three times, and the average was derived and taken as the diameter of the joint [45].

### 4.5. Arthritic Pain Evaluation

Arthritic pain was evaluated weekly with the static weight-bearing and PWT tests at the time points indicated in Figure 7. In rats, the weight distribution is typically symmetrical on both sides. Pain intensity was evaluated by assessing the disparity in weight bearing between the injured and uninjured hind limbs. A static weight-bearing test was performed with an Ugo Basile Incapacitance Tester [46]. Measurements were taken with the rat’s hind limbs positioned over the platforms while the rat remained stationary. The weight distribution of both hind limbs was recorded three times over a period of 5 s, and the following formula was used to calculate the limb weight distribution ratio (%).

$$Limb\;weight\;distribution\;ratio\left(\%\right)=\frac{injured\;limb\;load\left(g\right)\times 100}{\left(uninjured\;limb\;load\left(g\right)+injured\;limb\;load\left(g\right)\right)}$$

Mechanical allodynia was evaluated at similar time points (Figure 7) using a dynamic plantar aesthesiometer (Ugo Basile, Comerio, VA, Italy) with a stimulus increment rate of 2.5 g/s and a cutoff value of 50 g. Each rat underwent a 15-min adaptation period in a plastic cage. Subsequently, a filament was positioned on the palmar surface of the left hind paw and applied until the rat withdrew the hind paw. The pressure that caused paw withdrawal was recorded as the PWT. The result represents the mean of three independent measurements.

### 4.6. Histopathological Examination

The animals were euthanized on day 28 (Figure 7). The left knee joints were excised and fixed in 10% phosphate-buffered formalin for 3 days. Subsequently, the joints were decalcified with daily changes of 10% formic acid solution for 3 days. Following decalcification, the joints were sectioned and embedded in paraffin. The embedded joint was sectioned in the frontal plane at a 5-μm thickness. Two consecutive series of seven sections at 200-μm intervals near the center of the joint were collected and stained with hematoxylin and eosin for cytoarchitecture and Safranin O for the assessment of proteoglycans, chondrocytes, and type II collagen, respectively.

Histopathological scoring was conducted on the section located in the most central portion of each left knee joint. The morphology of the meniscus was used as an indicator of its proximity to the central part of the knee joint along the anterior–posterior axis. The section with the medial meniscus exhibiting an equilateral triangular shape is considered closest to the knee joint center; it was therefore selected for evaluation so that the center of the MTP can be consistently analyzed across all knee joints. After collagenase induction, the most severe lesion was typically found in the MTP as compared to the medial or the lateral femoral condyles, and the lateral tibial plateau. Each selected section represented the mid-coronal weight-bearing part of the entire joint in each rat.

Image evaluation was performed using NIS-Elements BR 5.42.02 64-bit analysis software (Nikon, Japan) with a Nikon Eclipse Ni microscope equipped with a Nikon DS-Ri2 camera. Parameters assessed included those recommended by the Osteoarthritis Research Society International (OARSI) for histological assessment in rats [47], Safranin O staining intensity [48], and chondrocyte density. The intensity of Safranin O staining is proportional to the proteoglycan content in the cartilage tissue when the proteoglycan content is not too low [49]. A brief overview of these parameters is provided in the Supplementary Information.

### 4.7. Statistical Analysis

Experimental results throughout this manuscript are expressed as means ± SEM. Statistical mean differences in continuous data between different groups were analyzed using one-way analysis of variance (ANOVA). Bonferroni correction was used for post hoc analysis. The minimal statistical significance was set at *p* < 0.05. All statistical analyses were performed using SPSS version 24 software (IBM, Armonk, NY, USA). Data were plotted using SigmaPlot 14.0 (Systat Software Inc., San Jose, CA, USA).

## 5. Conclusions

This study demonstrated the therapeutic effects of the intraarticular injection of synthetic deer antler peptides in alleviating OA symptoms and preserving cartilage integrity in a rat model of CIOA. The fact that these synthetic peptides have been shown in our previous experiments to stimulate the proliferation of human chondrocytes and osteoblasts in vitro argues for the great potential for this peptide cocktail to be a novel agent for the management of knee OA. Further studies focusing on elucidating the underlying mechanisms and ultimately conducting preclinical and clinical trials are warranted.

## Data Availability

Data or materials will be made available on request.

## Supplementary Materials

The following supporting information can be downloaded at: https://www.mdpi.com/article/10.3390/ijms25116041/s1.
