# Supplementary material for: The Therapeutic Potential of Intra-Articular Injection of Synthetic Deer Antler Peptides in a Rat Model of Knee Osteoarthritis
Source: Int J Mol Sci. 2024 May 30;25(11):6041. doi: 10.3390/ijms25116041 (PMC11172866; doi:10.3390/ijms25116041)
Supplement: Supplementary file 1 [file ijms-25-06041-s001.zip › ijms-3000012-supplementary.pdf]

Histopathological Scores:

1. Parameters based on the recommendations of the Osteoarthritis Research Society International (OARSI) for histological assessment in rats [1].

Cartilage Matrix Loss Width (CMLW):
The width of the regions displaying complete loss of the cartilage matrix was measured at three depths: surface (0% depth), mid-zone (50% depth), and tidemark (100% depth).

Significant Cartilage Degeneration Width (SCDW):
The width of the cartilage was measured, wherein 50% or more of its thickness (from the surface to the tidemark) was seriously compromised. When 50% of chondrocytes were absent or necrotic, along with or without loss of collagen matrix, a cartilage region was considered seriously compromised.

Total Cartilage Degeneration Score (TCDS):
The medial tibial plateau (MTP) was divided into three zones to assess the pathology in different load-bearing areas: zone 1 (medial edge of the joint), zone 2 (central area of the MTP), and zone 3 (adjacent to the cruciate ligaments). This parameter evaluates the overall cartilage pathology, with chondrocyte loss as the primary determinant of the score. Cartilage degeneration in each zone was scored based on the following criteria: 0 (no degeneration), 1 (minimal degeneration affecting 5–10% of the total projected cartilage area), 2 (mild degeneration affecting 11–25%), 3 (moderate degeneration affecting 26–50%), 4 (marked degeneration affecting 51–75%), and 5 (severe degeneration affecting greater than 75%). A 3-zone-sum for cartilage degeneration was calculated by adding the values obtained for each zone. The maximum 3-zone-sum of the medial tibia is 15.

2. Safranin O staining intensity:
Safranin O staining intensity was scored based on the MANKIN criteria for MTP [2]. This parameter was used to evaluate proteoglycan content in the cartilage tissue using the following criteria: 0 (normal staining, except for the surface zone), 1 (slight reduction, particularly in the superficial zone), 2 (moderate reduction, extending down to the mid-zone), 3 (severe reduction, involving the entire cartilage thickness), and 4 (absence of dye).

3. Chondrocyte density:
Chondrocytes were manually counted within the MTP, and the total area of the three load-bearing zones of the MTP was summed. The resulting numbers were divided by the total zone area to determine the chondrocyte density.

References

1. Gerwin, N.; Bendele, A. M.; Glasson, S.; Carlson, C. S., The OARSI histopathology initiative - recommendations for histological assessments of osteoarthritis in the rat. *Osteoarthritis Cartilage* **2010,** 18 Suppl 3, S24-34.
2. Pauli, C.; Whiteside, R.; Heras, F. L.; Nesic, D.; Koziol, J.; Grogan, S. P.; Matyas, J.; Pritzker, K. P.; D'Lima, D. D.; Lotz, M. K., Comparison of cartilage histopathology assessment systems on human knee joints at all stages of osteoarthritis development. *Osteoarthritis Cartilage* **2012,** 20, (6), 476-85.
